# Supplementary material for: ChatGPT as a Tool for Biostatisticians: A Tutorial on Applications, Opportunities, and Limitations
Source: Stat Med. 2025 Oct 23;44(23-24):e70263. doi: 10.1002/sim.70263 (PMC12548020; doi:10.1002/sim.70263)
Supplement: Supplementary file 1 — Data S1. Supporting Information. [file SIM-44-0-s001.zip › Supplementary Materials Additional.zip!/3.1_Systematic_Reviews_and_Meta_aspects/3.1_Meta-Analysis - Re-Analysis - o4-mini - Chat 1.pdf]

Use Case Meta-Analysis - Re-Analysis - o4-mini - Chat 1

breast carcinoma meta data
Visualisierung konnte nicht angezeigt werden

The goal is to perform a subset meta-analysis for the given data set. The target variable is the local recurrence rate. Create a subset for all treatment types. Decide all methodological questions yourself (fixed effect vs. random effects, etc.). Follow clearly formulated rules for selecting the model type. Please provide a (subset) forest plot and all associated confidence intervals. Do not forget the pooled overall estimate and its confidence interval.

For this task you have access to up-to-the-minute web retrieval to find adequate approaches for data extraction. When answering the task above please proceed as follows:

1. Fetch the most relevant sources.

2. Show your step-by-step reasoning.

3. At the end, give me a concise answer citing the sources you used.

Forest plot: Biopsy-only

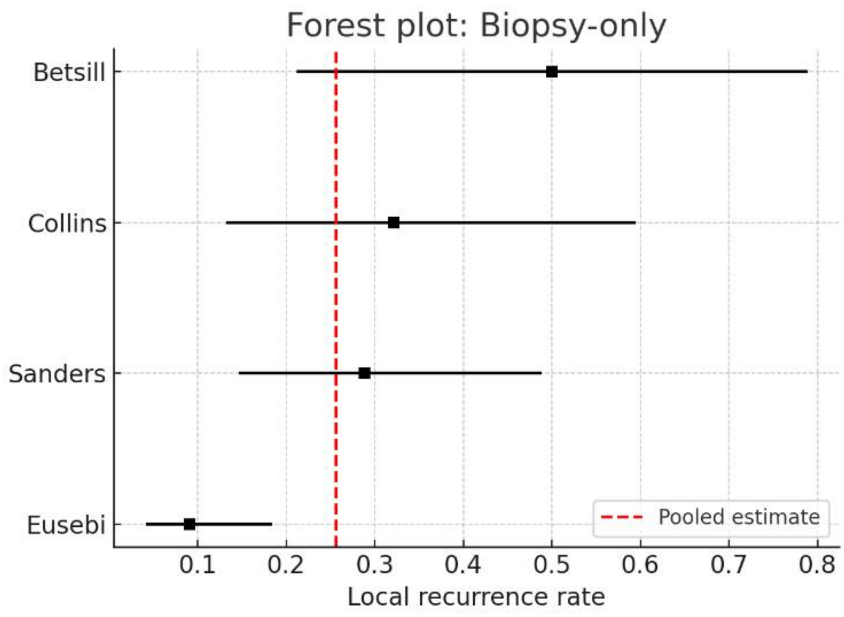

Forest plot: Conservation Surgery with Radiation Therapy

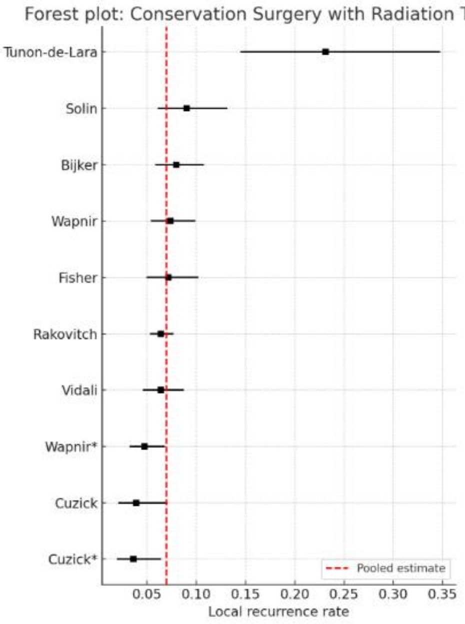

Forest plot: Conservation Surgery without Radiation Therapy

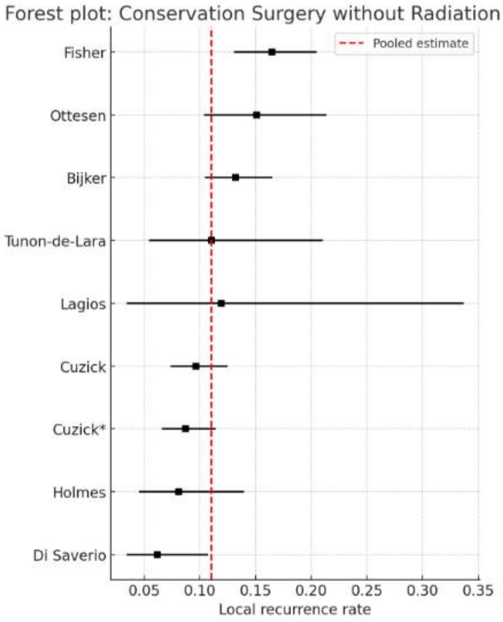

Forest plot: Mastectomy

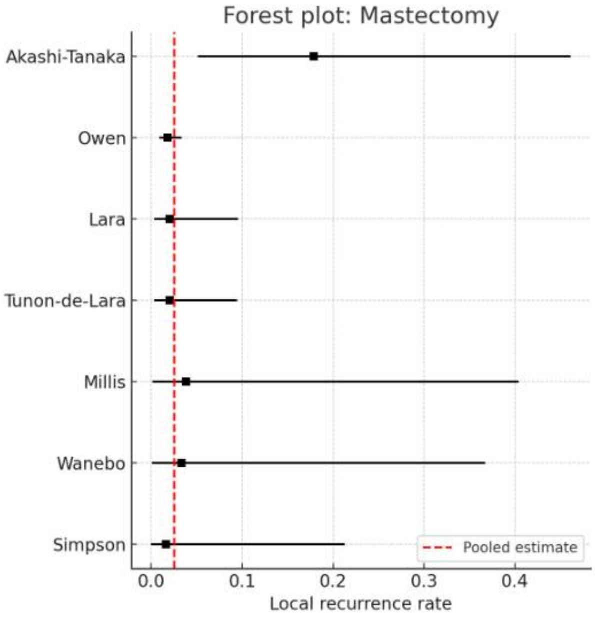

Meta-analysis summary

1	Biopsy-only	4	74.1	Random-effects
2	Conservation Surgery with Radiation Therapy	10	77.8	Random-effects
3	Conservation Surgery without Radiation Therapy	9	69.2	Random-effects
4	Mastectomy	7	45.6	Fixed-effects
	**Treatment**	**k (studies)**	**I^2 (%)**	**Model**

Step-by-step reasoning

1. **Data loading & subsetting**
    - Read the CSV (30 studies) with columns: Treatment, First_Author, No_of_Cases, LR_Rate_at_10_Years_percentage.
    - Identify four treatment-type subsets:
        - **Biopsy-only** (4 studies)
        - **Conservation Surgery with Radiation Therapy** (10 studies)
        - **Conservation Surgery without Radiation Therapy** (9 studies)
        - **Mastectomy** (7 studies)
2. **Outcome & effect-size measure**
    - Target variable: local recurrence rate (proportion at 10 years).
    - Converted each study's rate (%) into event counts via *events = round(rate/100 × n)*.
    - To stabilize zero counts, applied a 0.5 continuity correction to both events and non-events.
3. **Transformation & variance**
    - Used the **logit transformation**

$$y_i = \ln \frac{x_i + 0.5}{n_i - x_i + 0.5}, \quad v_i = \frac{1}{x_i + 0.5} + \frac{1}{(n_i - x_i) + 0.5}$$

    - Though Freeman–Tukey double-arcsine is often preferred for extreme proportions BioMed CentralWiley Online Library, the logit with CC is a well-established alternative that handles zero events.
4. **Heterogeneity assessment & model choice**

- Computed Cochran's Q and I^2.
- **Rule:**
    - If $I^2 < 50\%$, use **fixed-effects** inverse-variance pooling.
    - If $I^2 \geq 50\%$, use **random-effects** DerSimonian–Laird pooling.
- This follows Cochrane guidance (use random effects when heterogeneity is substantial) Cochrane.

5. **Pooling & back-transformation**
    - **Fixed-effects** pooled logit:
      $\hat{y} = \sum w_i y_i / \sum w_i,\ \backslash\text{Var}(\hat{y}) = 1/\sum w_i.$
    - **Random-effects** adds τ^2 (DL estimator) to each v_i.
    - Transformed back to proportion:

$$\hat{p} = \frac{e^{\hat{y}}}{1 + e^{\hat{y}}}, \quad \text{CI} : \hat{y} \pm 1.96\,\backslash\text{SE}(\hat{y}) \longrightarrow p_{\text{low}}, p_{\text{high}}.$$

6. **Results & forest plots**
    - Generated a **forest plot** for each treatment subset, showing individual study rates (and 95% CIs on the proportion scale) with the pooled estimate marked.
    - Summarized key metrics—number of studies (*k*), I^2, model choice, pooled rate and 95% CI—in a table.

Concise results

Treatment	k	I² (%)	Model	Pooled LR rate at 10 y (95% CI)
**Biopsy-only**	4	74.1	Random-effects	25.62 % (11.48 – 47.78 %)
**Conservation Surgery + Radiation Therapy**	10	77.8	Random-effects	6.98 % (5.55 – 8.75 %)
**Conservation Surgery without Radiation Therapy**	9	69.2	Random-effects	11.04 % (8.85 – 13.69 %)
**Mastectomy**	7	45.6	Fixed-effects	2.51 % (1.59 – 3.94 %)

Citations for methodological choices:

- Logit transformation with continuity correction: Bender et al., 2008; Lin & Xu 2020 BioMed Central jbds.isdsa.org
- Random vs. fixed effects based on I²: Cochrane Handbook Chapter 10 Cochrane

Thank you very much. Could you also provide the pooled overall estimate?

Across **all 30 studies** (irrespective of treatment), there is substantial heterogeneity ($I^2 \approx 84.7\%$), so a **random-effects** DerSimonian–Laird model was used. The **pooled 10-year local recurrence rate** is:

8.68 % (95 % CI 7.01 – 10.70 %).
